# Development and validation of a risk score to assist screening for acute HIV-1 infection among men who have sex with men

**DOI:** 10.1186/s12879-017-2508-4

**Published:** 2017-06-14

**Authors:** Maartje Dijkstra, Godelieve J. de Bree, Ineke G. Stolte, Udi Davidovich, Eduard J. Sanders, Maria Prins, Maarten F. Schim van der Loeff

**Affiliations:** 1 Academic Medical Center, Department of Infectious Diseases, University of Amsterdam, P.O. Box 22700, 1100DE, Amsterdam, the Netherlands; 2 Public Health Service of Amsterdam, Department of Infectious Diseases, Research and Prevention, P.O. Box 2200, 1000CE, Amsterdam, the Netherlands; 3 Amsterdam Institute for Global Health and Development, University of Amsterdam, P.O. Box 22700, 1100DE, Amsterdam, the Netherlands; 4 Kenya Medical Research Institute, Centre for Geographic Medicine Research –Coast, P.O. Box 230, Kilifi, Kenya; 5 Nuffield Department of Clinical Medicine, University of Oxford, Oxford, OX3 7BN UK

**Keywords:** Acute HIV-1 infection, Screening tool, MSM, Risk score, Diagnosis

## Abstract

**Background:**

Early treatment of acute HIV-1 infection (AHI) is beneficial for patients and could reduce onward transmission. However, guidelines on whom to test for AHI with HIV-1 RNA testing are lacking.

**Methods:**

A risk score for possible AHI based on literature and expert opinion – including symptoms associated with AHI and early HIV-1 – was evaluated using data from the Amsterdam Cohort Studies among men who have sex with men (MSM). Subsequently, we optimized the risk score by constructing two multivariable logistic regression models: one including only symptoms and one combining symptoms with known risk factors for HIV-1 seroconversion, using generalized estimating equations. Several risk scores were generated from these models and the optimal risk score was validated using data from the Multicenter AIDS Cohort Study.

**Results:**

Using data from 1562 MSM with 175 HIV-1 seroconversion visits and 17,271 seronegative visits in the Amsterdam Cohort Studies, the optimal risk score included four symptoms (oral thrush, fever, lymphadenopathy, weight loss) and three risk factors (self-reported gonorrhea, receptive condomless anal intercourse, more than five sexual partners, all in the preceding six months) and yielded an AUC of 0.82. Sensitivity was 76.3% and specificity 76.3%. Validation in the Multicenter AIDS Cohort Study resulted in an AUC of 0.78, sensitivity of 56.2% and specificity of 88.8%.

**Conclusions:**

The optimal risk score had good overall performance in the Amsterdam Cohort Studies and performed comparable (but showed lower sensitivity) in the validation study. Screening for AHI with four symptoms and three risk factors would increase the efficiency of AHI testing and potentially enhance early diagnosis and immediate treatment.

**Electronic supplementary material:**

The online version of this article (doi:10.1186/s12879-017-2508-4) contains supplementary material, which is available to authorized users.

## Background

Acute HIV-1 infection is the phase of HIV-1 disease immediately after infection that is characterized by an initial burst of viremia; although anti-HIV-1 antibodies are undetectable, HIV-1 RNA or p24 antigen are present [1, 2]. Identifying patients with AHI is important for three reasons. First, patients benefit from immediate start of combination antiretroviral therapy (cART) during the early phase of infection [3–5]. Second, starting cART soon after infection could have a significant impact on the ongoing HIV-1 epidemic [6–9]. Very high viral loads during AHI and continued high-risk behavior before diagnosis are major determinants of transmission [1, 10, 11]. Third, patients who start cART during AHI may offer insight into the potential for post treatment control of HIV-1 infection [12–15].

The US Centers for Disease Control & Prevention included HIV-1 RNA testing in their guidelines for detecting AHI [16]. The recent development of point-of-care HIV-1 RNA tests have made the prompt diagnosis of AHI possible at the point-of-care [17] and offers a unique opportunity to increase the number of HIV-1 infections diagnosed during the acute phase, potentially even before p24 antigen is detectable. However, these tests are expensive and guidelines on whom to test for AHI are lacking.

Criteria for testing might include symptoms of AHI. Forty to 90% of patients with AHI experience symptoms, but these symptoms are transient and often nonspecific [2, 18, 19]. Timely recognition of individuals in the early weeks of infection with HIV-1 RNA or p24 antigen tests may change the course of infection [20]. A suitable screening tool used to select patients for AHI testing would restrict testing to a limited number of individuals, while detecting a large proportion of all AHI cases. Recent studies from sub-Saharan Africa reported the development of risk scores for AHI screening [21–23] with good performance among men who have sex with men (MSM) [21]. It remains to be elucidated whether risk scores developed in recourse-limited settings could be translated into a different context, where different risk behavior and HIV-1 subtype may influence factors included in the risk scores [21, 24]. Risk scores for AHI detection in high-income countries have been previously developed [25, 26], but a risk score for use for the symptomatic patient at the point-of-care is lacking.

As part of a recently launched initiative aiming to curb the epidemic at the city level, the HIV Transmission Elimination AMsterdam initiative (H-TEAM) [27], an AHI testing strategy was developed in order to identify individuals with AHI and start immediate treatment. This strategy included a risk score for possible AHI that weighs symptoms in combination with sexual risk behavior. This risk score was implemented as an interactive online screening tool for MSM through a dedicated website [28], used by MSM and health providers at the sexually transmitted infections (STI) clinic of the Public Health Service of Amsterdam (GGD). In the present study, we aimed to assess whether such a risk score could be useful for AHI screening in a public health setting. We aimed to evaluate the performance of the AHI risk score using data from the Amsterdam Cohort Studies (ACS) and to assess whether the H-TEAM risk score could be optimized in the Amsterdam setting. We validated the optimal risk score using data from the Multicenter AIDS Cohort Study (MACS) in the USA.

## Methods

### Initial development of a risk score for AHI

A recent meta-analysis reviewed 21 studies on acute and early HIV-1 infection and showed that seventeen symptoms and seven signs were associated with acute or early HIV-1 infection [18]. The symptoms are summarized in Table 1. A pragmatic risk score for possible AHI was developed based on peer-reviewed literature and comprehensive consultations with experts. This resulted in an individual risk score for MSM which is equal to the sum of scores for a list of seventeen symptoms, whereby presence of a symptom either yields a score of one (fatigue, genital warts, headache, myalgia, nausea, night sweats, pharyngitis, or rash) or a score of two (arthralgia, diarrhea, fever, genital ulcers, lymphadenopathy, oral thrush, oral ulcers, vomiting, or weight loss). These scores were based on the positive likelihood ratio for each symptom in association with acute or early HIV-1 infection, as reported by Wood et al. [18]. We translated this list of symptoms into a self-administered online screening tool by which patients could refer themselves to the STI clinic of the Public Health Service of Amsterdam (GGD) for AHI testing, including a point-of-care HIV-1 RNA test. The initial pragmatic risk score (risk score **A**) was: possible AHI is present if an individual reports condomless anal intercourse (CLAI) in the preceding three months and has a risk score of at least two using the above mentioned symptom list.

**Table 1:** Symptoms associated with AHI and questions in ACS and MACS

Symptom^a^	ACS questionnaire	MACS questionnaire
Arthralgia		x
Diarrhea	x	x
Fatigue	x	x
Fever	x	x
Genital ulcers	x	x
Genital warts	x^b^	x
Headache	x	x
Lymphadenopathy	x	x
Myalgia/arthralgia	x^c^	x^d^
Nausea	x	x^e^
Night sweats	x	x
Oral thrush	x	x
Oral ulcers	x^b,f^	x^f^
Pharyngitis	x^g^	x^h^
Rash	x	x
Vomiting	x	x^e^
Weight loss	x	x

*ACS* Amsterdam Cohort Studies, *AHI* acute HIV-1 infection, MACS Multicenter AIDS Cohort Study

^a^Resulting from a meta-analysis based on 21 studies on acute and early HIV infection [18]

^b^Asked from 2006 onwards

^c^Data on myalgia only

^d^Asked through 1989 (muscle/joint pain), and from 2001 onwards question changed to muscle pain/weakness

^e^Data on nausea/vomiting combined, asked from 2001 onwards

^f^Data on labial (ACS) or facial (MACS) herpes

^g^Data on sore throat

^h^Data on sore throat/sore mouth combined, asked through 1989

### Performance of the initial risk score in Amsterdam cohort studies

To validate our initial risk score (risk score **A**) we used data from the ACS. This is an open, prospective cohort study among MSM in Amsterdam, initiated in 1984 aiming to investigate the epidemiology, natural history, and pathogenesis of HIV-1, and to evaluate the effect of interventions [29]. Until 1995, men of all age groups were eligible to participate if they had had at least two male sexual partners in the preceding six months. From 1995 to 2004, men aged thirty or less with at least one male sexual partner in the preceding six months could enter the study. From 2005 onwards, men of all age groups with at least one male sexual partner in the preceding six months could participate. Participation is voluntary, and written informed consent is obtained from every participant at enrollment.

Study participants visited the GGD every six months to complete a self-administered questionnaire on risk behavior in the preceding six months [30]. A face-to-face standardized interview with a study nurse captured symptoms experienced since last study visit. All except four of the symptoms in our initial risk score were included in the standardized interview; there was no question on arthralgia, and questions on oral ulcers and genital warts were only included in the interview from 2006 onwards. Furthermore, there was no question on pharyngitis, but sore throat was included. Physical examination was not performed. Each visit, participants provided a blood sample for HIV-1 testing by consecutive generations of commercially available screening assays to detect HIV-1 antibodies and for storage. HIV-1 test results were confirmed by Western blot analysis. A seroconversion visit was defined as the visit with the first positive HIV-1 test result after a visit with an HIV-1 seronegative result. For this study, we included MSM who were HIV-1 negative at study entry and had had at least one follow-up visit between October 1984 and December 2009. Any visit with an interval of more than twelve months following the preceding visit was excluded from the analyses.

Points were assigned to each of the symptoms included in the ACS questionnaire according to the scoring procedure of the initial risk score (**A**). Oral ulcers and genital warts were excluded from the analyses due to large numbers of missing data (questions only asked from 2006 onwards). In total, fourteen symptoms were included in the analysis of this risk score. Reported CLAI was a condition in this risk score and a pre-specified cutoff of two was used, both consistent with the initial risk score (**A**).

### Optimizing the risk score

We aimed to improve the initial risk score by analyzing the association of symptoms with HIV-1 seroconversion in the ACS. Analysis was done with logistic regression using generalized estimating equations, with an exchangeable covariance matrix and robust standard errors to adjust for multiple observations per participant. We calculated odds ratios (OR) for HIV-1 seroconversion for each symptom in our initial risk score, using univariable analysis. A multivariable logistic regression model including the fourteen symptoms was constructed. We created a risk score in which each symptom received points equal to its beta coefficient (β, natural log of the adjusted odds ratio) in the multivariable model, rounded to the nearest decimal [23]. CLAI was a condition in this risk score (risk score **B**), consistent with the initial risk score. Because not all seroconverters reported CLAI, we evaluated performance of this risk score also without CLAI as a condition (risk score **C**).

Next, we developed a risk score in which we combined symptoms with risk factors reported at a seroconversion visit. An earlier analysis of the ACS data found that self-reported gonorrhea, receptive CLAI, more than five sexual partners (all in the preceding six months) and lower educational level were associated with HIV-1 seroconversion in this study population [30]. After consultation with clinical experts we decided not to incorporate lower educational level in the risk score, as this was considered inappropriate. The three remaining risk factors were included in a multivariable model, thus including fourteen symptoms and three risk factors, and a risk score with all variables scored as the β rounded to the nearest decimal was generated (risk score **D**). In order to simplify the risk score, performance of a risk score including only variables that were statistically significant (*p* < 0.05) in the combined symptom and risk factor multivariable model was also evaluated (risk score **E**) [23].

We compared seroconversion visits with seronegative visits to evaluate the performance of all risk scores generated. We used receiver operating characteristics (ROC) curves to calculate the area under the curve (AUC). To evaluate sensitivity and specificity at a consistent cutoff for all risk scores, the Youden-index [31] was calculated for each risk score, and sensitivity and specificity were defined at this cutoff. The optimal risk score was defined as good overall performance (i.e. high AUC) and suitable to use as a screening tool (i.e. a limited number of symptoms and risk factors).

To study differences in symptoms and risk factors by age and over time we categorized age at time of the study visit (≤24, 25–34, 35–44, and ≥45 years of age) and calendar year of visit (<1990, 1990–1994, 1995–1999, 2000–2004, 2005–2009). We added these categorical age and calendar year variables to the multivariable model including fourteen symptoms and three risk factors. The HIV-1 epidemic was over its peak after 1990 [30], therefore we performed a sensitivity analysis by including only visits from 1990 onwards in the multivariable model. In another sensitivity analysis, we included educational level in the multivariable model.

It is possible that infection occurred at the visit before the seroconversion visit, therefore we explored the prevalence of reported symptoms at the last HIV-1 negative visit prior to seroconversion. Lastly, we evaluated the interval between the seroconversion visit and the previous visit for all seroconverters.

The ACS dataset had missing values for several variables, varying between zero and 2546 out of a total of 17,446 observations. For six variables no data were missing, for fourteen variables less than 1% of records had missing data, and for four variables data were missing from more records, the largest missing number being 2546 for the variable gonorrhea. We used the last observation carried forward method to impute missing values. We only imputed values of a variable if the value of the variable of the visit immediately preceding was available. We also conducted complete case analyses using only records without missing values.

### Validation of the risk score

In order to validate the optimal risk score data from the MACS were used. The MACS is an ongoing prospective study in homosexual and bisexual men located in Baltimore, Chicago, Pittsburgh and Los Angeles (USA) [32]. Study visits were scheduled every six months. We used the Public Data Sets containing data from 1984 to October 2010 (version P23, released on 15 October 2014 [33]). Participants who were HIV-1 negative at baseline and had at least one follow-up visit were included in the analyses. Participants who seroconverted for HIV-1 infection during follow-up, but did not have a registered seroconversion visit or date of seroconversion (*n* = 39) were excluded from the analyses. The MACS Public Data Sets did not include date of visit, so visits were excluded when a participant had skipped two or more previous visits (any visit above twelve months following the previous visit, assuming that the protocol of semiannual visits was precisely followed). The remaining dataset, including 63,618 visits, contained large numbers of missing values for all variables, in part because the questionnaires varied by study visit: questions on fever (5883 missings) and lymphadenopathy (5881 missings) were asked from 1986 onwards, oral thrush (24,013 missings) was asked from 1990 onwards, and weight loss (525 missings), gonorrhea (562 missings), number of male sex partners (8850 missings) and receptive CLAI (21,344 missings) were included in the questionnaire through all years. We used the last observation carried forward method to impute missing values, but only if the value of the variable of the visit immediately preceding was available.

### Post-test probability of disease

To evaluate the clinical relevance of the optimal risk score, the post-test probability of disease (PTPD; i.e. the likelihood of having AHI when having a score above the risk score cutoff [34]) was estimated. Prevalence of AHI has not been evaluated in the Netherlands, therefore AHI prevalence from a recent study in the USA among sexually active MSM was used (AHI prevalence: 151/44955, 0.34% [35]).

Statistical analyses were conducted using Stata version 13.1 (StataCorp, College Station, Texas, USA).

## Results

In total, 1562 MSM who were HIV-1 negative at enrollment in the ACS were included in the analyses. They accounted for 175 HIV-1 seroconversion visits and 17,217 seronegative visits for the period 1984 through 2009. The median number of visits was 7 (interquartile range (IQR) 4–13). Median age at study entry was 34.4 years (IQR 29.4–41.4).

### Performance of the initial risk score in Amsterdam cohort studies

**Fig. 1:**
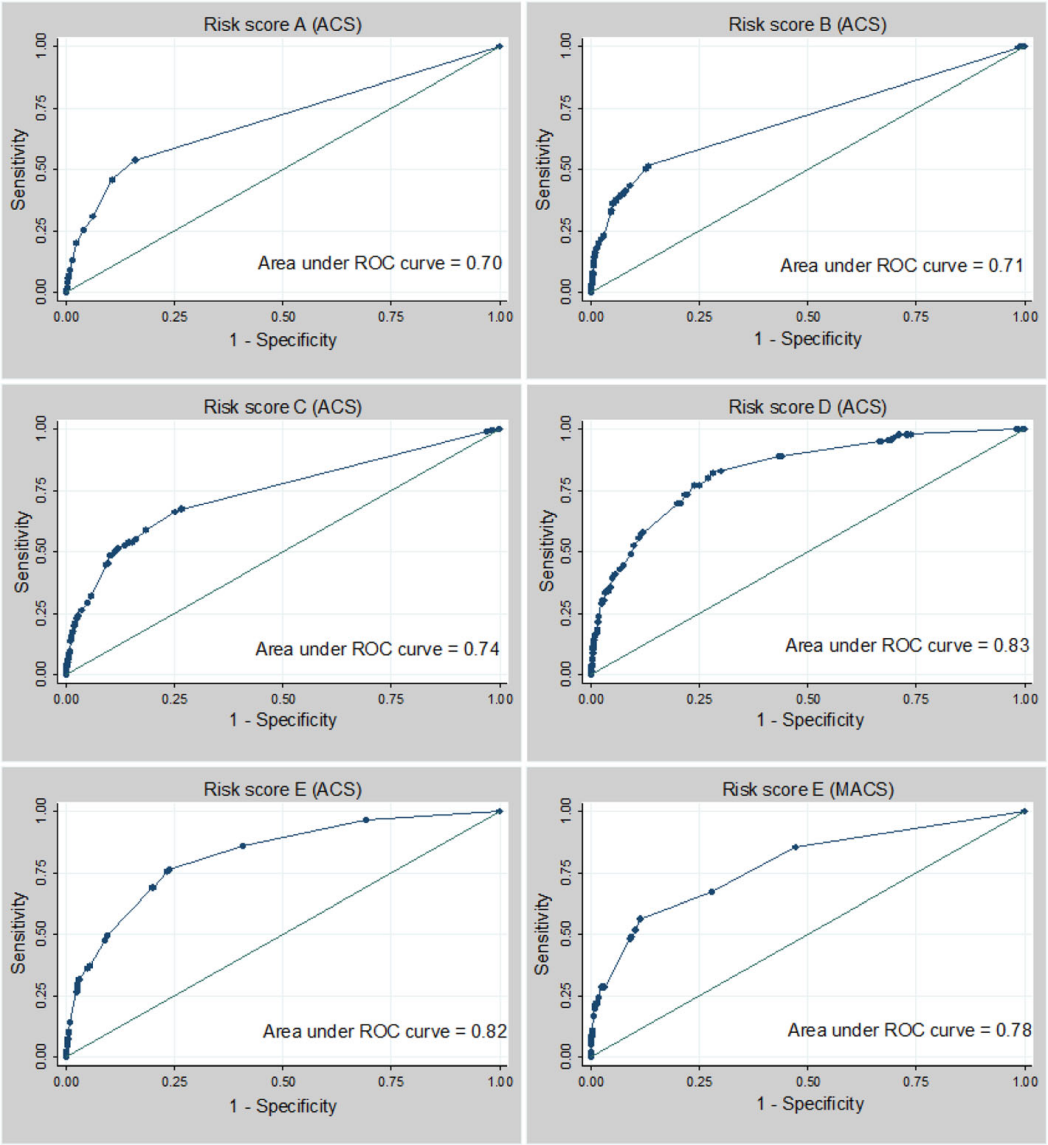
**a**-**e**. ROC curves of risk scores in ACS and validation in MACS. *ACS*, Amsterdam Cohort Studies; *MACS*, Multicenter AIDS Cohort Study; *ROC*, receiver operating characteristics

In the ACS, the overall AUC for the initial risk score was 0.70 (risk score **A**). With the pre-defined risk score cutoff of two, sensitivity was 45.7% (95% confidence interval (CI) 38.2–53.4) and specificity 89.5% (95%CI 89.1–90.0). By applying this risk score to ACS study participants, 10.8% would be indicated for AHI testing, identifying 45.7% of cases (Table 2 and Fig. 1a).

**Table 2:** Performance of several risk scores for AHI^a^

Column 1	2	3	4	5	6	7	8
Risk score	Cutoff^b^	Seroconversion visits among visits with a score of at least the cutoff^c^	Seroconversion visits among visits with a score below the cutoff^c^	Sensitivity % (95% CI)	Specificity % (95% CI)	Overall AUC (95% CI)	% to be tested
Development of a risk score in Amsterdam Cohort Studies							
A. Initial risk score^d,e^ -CLAI a condition^f^	2	80/1888	95/15555	45.7 (38.2–53.4)	89.5 (89.1–90.0)	0.70 (0.66–0.74)	10.8
B. 14 symptoms scored as beta coefficient in symptom based model^d,g^ -CLAI a condition^f^	0.1	90/2342	85/15101	51.4 (43.8–59.0)	87.0 (86.4–87.5)	0.71 (0.67–0.74)	13.4
C. 14 symptoms scored as beta coefficient in symptom based model^g,h^ -CLAI not a condition	0.2	116/4430	59/13012	66.3 (58.8–73.2)	75.0 (74.4–75.7)	0.74 (0.70–0.78)	25.4
D. 14 symptoms and 3 risk factors, scored as beta coefficient in combined model^i,j^	1.3	111/4334	24/10858	82.2 (74.7–88.3)	72.0 (71.2–72.7)	0.83 (0.80–0.87)	28.5
E. 4 symptoms and 3 risk factors scored as beta coefficient in combined model^k,j^	1.5	103/3675	32/11517	76.3 (68.2–83.2)	76.3 (75.6–77.0)	0.82 (0.79–0.86)	24.2
Validation of optimal risk score in Multicenter AIDS Cohort Study							
E. 4 symptoms and 3 risk factors^k^, scored as beta coefficient	1.5	77/3779	60/29274	56.2 (47.5–64.7)	88.8 (88.4–89.1)	0.78 (0.74–0.82)	11.4

*AHI* acute HIV-1 infection, *CI* confidence interval, *CLAI* condomless anal intercourse

^a^Based on imputed data using the last observation carried forward method

^b^Based on Youden-index, with the exception for risk score A: pre-defined cutoff was 2

^c^Due to missing values, the denominators of columns 3 and 4 do not add up to 17,446. Data were missing for the following variables: fever, 1 missing; genital ulcers, 3 missings; gonorrhea, 2072 missings; receptive condomless anal intercourse, 162 missings; >5 sexual partners, 56 missings.

^d^Due to 3 records with missing values, these risk score evaluations are based on 17,443 records

^e^14 symptoms scored as case definition, but genital warts and oral ulcers not included due to large number of missing variables; score of 1: fatigue, headache, myalgia, nausea, night sweats, pharyngitis, or rash; score of 2: diarrhea, fever, genital ulcers, lymphadenopathy, oral thrush, oral ulcers, vomiting, or weight loss

^f^112/175 seroconverters reported condomless anal intercourse in the preceding 6 months

^g^In a multivariable model including 14 symptoms (Table 3, column 6): diarrhea, fatigue, fever, genital ulcers, headache, lymphadenopathy, myalgia, nausea, night sweats, oral thrush, rash, sore throat, vomiting, weight loss

^h^Due to 4 records with missing values, these risk score evaluations are based on 17,442 records

^i^In a multivariable model including 14 symptoms and 3 risk factors (Table 3, column 8): diarrhea, fatigue, fever, genital ulcers, headache, lymphadenopathy, myalgia, nausea, night sweats, oral thrush, rash, sore throat, vomiting, weight loss, and in the preceding 6 months: gonorrhea, receptive condomless anal intercourse, >5 sexual partners

^j^Due to 2254 records with missing values, these risk score evaluations are based on 15,192 records

^k^Fever, lymphadenopathy, oral thrush, weight loss, and in the preceding 6 months: gonorrhea, receptive condomless anal intercourse, >5 sexual partners

### Optimizing the risk score

Prevalence of self-reported symptoms in the preceding six months is shown in Table 3. In univariable analysis, all fourteen symptoms were significantly associated with HIV-1 seroconversion. Self-reported oral thrush (OR 10.8, 95%CI 3.8–30.7), fever (OR 7.2, 95%CI 5.4–9.8), and lymphadenopathy (OR 7.2, 95%CI 4.7–10.9) were most strongly correlated with seroconversion. In a multivariable logistic regression model including all fourteen symptoms, oral thrush (adjusted odds ratio (aOR) 5.0, 95%CI 1.5–16.3), fever (aOR 5.0, 95%CI 3.5–7.1), lymphadenopathy (aOR 3.3, 95%CI 2.1–5.4) and genital ulcers (aOR 2.6, 95%CI 1.3–5.0) remained statistically significant.

**Table 3:** Prevalence of symptoms and risk factors and their association with HIV-1 seroconversion^a^

Column 1	2		3		4	5	6	7	8
Symptom	Seroconversion visit *n*%		HIV negative visit *n*%		OR (95% CI)	Symptom based model aOR (95% CI)^b^	Beta coefficient^c,d^	Combined model aOR (95% CI)^e^	Beta coefficient^c,f^
Total^g^	175		17,271						
Symptom									
Diarrhea	30	17.1	1011	5.9	3.3 (2.2–4.8)	1.3 (0.8–2.1)	0.3	1.5 (0.9–2.4)	0.4
Fatigue	42	24.0	1867	10.8	2.6 (1.8–3.6)	1.2 (0.8–1.9)	0.2	1.4 (0.9–2.2)	0.3
Fever	76	43.4	1623	9.4	7.2 (5.4–9.8)	5.0 (3.5–7.1)^h^	1.6	4.7 (3.2–7.0)^h^	1.6
Genital ulcers	11	6.3	324	1.9	3.5 (1.9–6.4)	2.6 (1.3–5.0)^h^	0.9	1.6 (0.6–4.0)	0.5
Headache	18	10.3	980	5.7	1.9 (1.2–3.1)	0.7 (0.4–1.3)	−0.3	0.8 (0.4–1.5)	−0.2
Lymphadenopathy	27	15.4	418	2.4	7.2 (4.7–10.9)	3.3 (2.1–5.4)^h^	1.2	4.4 (2.6–7.3)^h^	1.5
Myalgia	25	14.3	869	5.0	3.1 (2.0–4.7)	1.1 (0.7–1.9)	0.1	1.4 (0.8–2.4)	0.3
Nausea	18	10.3	526	3.1	3.6 (2.2–5.8)	0.8 (0.4–1.7)	−0.2	0.9 (0.4–1.9)	−0.1
Night sweats	32	18.3	832	4.8	4.3 (3.0–6.4)	1.2 (0.8–2.0)	0.2	1.2 (0.7–2.1)	0.2
Oral thrush	4	2.3	36	0.2	10.8 (3.8–30.7)	5.0 (1.5–16.3)^h^	1.6	5.6 (1.4–22.3)^h^	1.7
Rash	11	6.3	564	3.3	2.0 (1.1–3.6)	1.2 (0.6–2.4)	0.2	0.6 (0.3–1.4)	−0.5
Sore throat	31	17.7	1316	7.6	2.6 (1.7–3.8)	1.0 (0.7–1.6)	0.0	0.9 (0.5–1.4)	−0.2
Vomiting	17	9.7	336	2.0	5.3 (3.2–8.8)	1.8 (0.9–3.8)	0.6	1.4 (0.6–3.3)	0.4
Weight loss	11	6.3	205	1.2	5.5 (2.9–10.2)	1.9 (1.0–3.9)	0.7	2.4 (1.1–5.2)^h^	0.9
Risk factor									
Gonorrhea^i,j^	12	6.9	181	1.1	6.5 (3.6–12.0)			4.9 (2.3–10.2)^h^	1.6
Receptive CLAI^i^	97	55.4	5651	32.7	2.7 (2.0–3.7)			3.0 (2.1–4.2)^h^	1.1
>5 sexual partners^i,k^	100	57.1	7659	44.4	2.4 (1.7–3.4)			2.5 (1.7–3.6)^h^	0.9

*aOR* adjusted odds ratio. *CI* confidence interval, *CLAI* condomless anal intercourse, *OR* odds ratio

^a^In Amsterdam Cohort Studies, based on imputed data using the last observation carried forward method

^b^14 symptoms in column 1 were included in a multivariable logistic regression model using generalized estimating equations

^c^Natural log of the adjusted odds ratio, rounded to one decimal

^d^Based on the adjusted odds ratios of the symptom-based model (column 5)

^e^14 symptoms and 3 risk factors in column 1 were included in a multivariable logistic regression model using generalized estimating equations

^f^Based on the adjusted odds ratios of the combined symptom and risk factor model (column 7)

^g^Due to missing values, the denominator of columns 2 and 3 are sometimes not equal to 175 respectively 17,271. Data missing for the following variables: fever, 1 missing; genital ulcers, 3 missings; gonorrhea, 2072 missings; receptive condomless anal intercourse, 162 missings; >5 sexual partners, 56 missings

^h^Significantly associated with HIV-1 seroconversion

^i^In the preceding 6 months

^j^Self-reported

^k^Only male sex partners, compared to ≤5 sexual partners

Three risk factors (self-reported gonorrhea, receptive CLAI, more than five sexual partners, all in the previous six months) for HIV-1 seroconversion previously described in the ACS [30] were significantly associated with seroconversion in univariable analysis (Table 3, column 4). The results of the combined multivariable model with fourteen symptoms and three risk factors are shown in Table 3, column 7. In this combined multivariable model, oral thrush (aOR 5.6, 95%CI 1.4–22.3), fever (aOR 4.7, 95%CI 3.2–7.0), lymphadenopathy (aOR 4.4, 95%CI 2.6–7.3), weight loss (aOR 2.4, 95%CI 1.1–5.2), self-reported gonorrhea (aOR 4.9, 95%CI 2.3–10.2), receptive CLAI (aOR 3.0, 95%CI 2.1–4.2), and more than five sexual partners (aOR 2.5, 95%CI 1.7–3.6) (all in the preceding six months) remained statistically significant.

The overall AUC was 0.71 for a risk score with points assigned to each symptom according to the β in a symptom based model in which CLAI was a condition (risk score **B**, Fig. 1b). The same risk score, but without CLAI as a condition (risk score **C**) resulted in an overall AUC of 0.74 (Fig. 1c).

In a risk score including fourteen symptoms and also three risk factors, the overall AUC was 0.83 (risk score **D**, Fig. 1d). A simplified risk score including only variables statistically significantly associated with HIV-1 seroconversion in the multivariable model yielded an AUC of 0.82 (risk score **E**). At the risk score cutoff of 1.5 (Youden index), sensitivity was 76.3% (95%CI 68.2–83.2) and specificity was 76.3% (95%CI 75.6–77.0). Applying this risk score to ACS participants, 24.2% would be indicated for AHI testing, identifying 76.3% of cases. Choosing a less stringent cutoff of 1.1 would result in sensitivity of 85.9% but lower the sensitivity to 59.3% (Fig. 1e). Risk score **E** was defined as the optimal risk score because of a relatively high AUC in combination with simplicity of the risk score (based on only seven variables, all statistically significant in the multivariable model) and therefore suitable for implementation as a screening tool.

In a multivariable logistic regression model that also included age (categorized) and calendar year of visit (categorized), the β of symptoms and risk factors were comparable to the original model (data not shown). An analysis limited to visits from 1990 onwards resulted in an increase of the aOR of myalgia (aOR 2.6, 95%CI 1.4–4.8). Applying the optimal risk score (**E**) to data from 1990 onwards did not change the performance of the risk score (AUC 0.82), and very similar outcomes (sensitivity, specificity and AUC) were observed when myalgia was added to this risk score (data not shown). Adding educational level to the multivariable model did not result in major changes in the β of symptoms and risk factors, but lower educational level itself was significantly associated with HIV-1 seroconversion (β 0.7, data not shown). A risk score including the variables from the optimal risk score (**E**) and also educational level showed similar performance (AUC 0.83) compared to the optimal risk score (data not shown). Complete case analyses did not show important differences in outcomes compared to the analyses based on imputed data, but weight loss was no longer significantly associated with HIV-1 seroconversion in the combined symptom and risk factor model (Additional file 1: Table S1 and S2).

For participants who seroconverted during follow-up, prevalence of reported symptoms was consistently lower at the last HIV-1 negative visit preceding seroconversion. There was one participant who seroconverted in which the time interval between the first seropositive visit and the last seronegative visit was more than nine months. Additionally, retrospective HIV-1 RNA testing of stored blood samples among seroconverters showed that 2/175 seroconverters in this study had a positive RNA test result at the visit preceding their seroconversion visit (data not shown).

### Validation of the risk score

The optimal risk score (**E**) was validated in the MACS, with data on 3751 MSM with 491 seroconversion visits and 63,127 seronegative visits. Due to a large number of missings for all variables, the evaluation of the optimal risk score was based on 137 seroconversion visits and 32,916 seronegative visits. The overall AUC was 0.78 (Fig. 1e). Using 1.5 as a cutoff, sensitivity was 56.2% (95%CI 47.5–64.7) and specificity 88.8% (95% CI 88.4–89.1). 11.4% would be indicated for RNA testing, which would identify 56.0% of cases. Table 4 shows sensitivity and specificity at several cutoff levels for this risk score in the MACS.

**Table 4:** Validation of optimal risk score E.^a^ in MACS, 1984–2010

Cutoff^b^	Sensitivity %	Specificity %	Positive LR	Negative LR	% to be tested
≥ 0.9	85.4	53.0	1.8	0.3	47.2
≥ 1.1	67.2	72.3	2.4	0.5	27.9
≥ 1.5	56.2	88.8	5.0	0.5	11.4
≥ 1.6	51.8	89.9	5.1	0.5	10.3
≥ 1.7	48.9	90.7	5.3	0.6	9.5
≥ 1.8	48.9	90.9	5.4	0.6	9.3
≥ 2.0	48.8	91.1	5.4	0.6	9.1
≥ 2.4	28.5	97.1	9.8	0.7	3.0

*LR* likelihood ratio, *MACS* Multicenter AIDS Cohort Study

^a^Including 4 symptoms and 3 risk factors (Table 3, row 7): fever, lymphadenopathy, oral thrush, weight loss, gonorrhea, receptive condomless anal intercourse, >5 sexual partners

^b^Cutoff levels were defined by each possible sum of the score of symptoms and risk factors, based on the beta coefficient (Table 3, column 8)

### Post-test probability of disease

Risk score **E** was further used to estimate the PTPD in the ACS. Using an AHI prevalence of 0.34% from a similar MSM population [35] (i.e. the probability of AHI for an individual before applying the risk score) and a risk score cutoff of 1.5, the PTPD (i.e. the probability of AHI after having an AHI testing advice) was 1.09%. The positive likelihood ratio at this cutoff was 3.2.

## Discussion 

We aimed to assess whether a risk score could be useful for AHI screening. Several risk scores were generated and performance was assessed using data from MSM cohorts in Amsterdam (ACS) and the USA (MACS). The initial risk score (**A**) – generated by expert opinion, including fourteen symptoms and CLAI as a condition – had a weak overall performance in the ACS. Other risk scores including only symptoms also had a weak performance. The addition of three risk factors for HIV-1 seroconversion to the risk score resulted in good overall performance in the ACS.

The optimal risk score included oral thrush (β 1.7), fever (β 1.6), lymphadenopathy (β 1.5), weight loss (β 1.9), self-reported gonorrhea (β 1.6), receptive CLAI (β 1.1), and more than five sexual partners (β 0.9) (all in the preceding six months) (risk score **E**). Using this risk score in the ACS, with a cutoff of 1.5, 24% of men would be assigned as having possible AHI and indicated for HIV-1 RNA testing. This would identify 76% of seroconverters, with a PTPD of 1.09%. The overall AUC was 0.82. Validation of this risk score in the MACS resulted in a similar AUC (0.78) but lower sensitivity (56%). These outcomes are comparable with previous studies [21–23, 36]. A study including data of AHI patients from four African sites described a risk score (including age 18–29 years, fever, diarrhea, fatigue, body pains, sore throat and genital ulcer disease) resulting in an overall AUC of 0.78, and among Kenyan MSM specifically, the AUC was 0.89, sensitivity 90% and specificity 74% [21].

This study has several limitations. First, in both ACS and MACS the interval of six months between study visits was relatively long. Questionnaires in both cohorts included questions on symptoms and risk behavior in the preceding six months. These were reported at seroconversion visits, but it is unclear whether symptoms occurred during AHI. Twenty-nine percent of seroconverters in the ACS did not report any symptom in the preceding six months (data not shown). AHI might have been asymptomatic [24], associated with nonspecific symptoms [18, 37] or symptoms might have been underreported due to recall bias. A multicenter study from Africa showed that prevalence of reported AHI symptoms of patients evaluated within six weeks following infection was higher as compared to patients who were evaluated later [38]. This may also explain the relatively low sensitivity observed in the present study. Thus, this relatively long interval between study visits may have led to an underestimation of the performance of the risk scores. Furthermore, the period of acute infection might have been occurring at the previous study visit, when antibodies were not yet detectable, rather than at the seroconversion visit. However, in the ACS, fewer symptoms were reported at last HIV-1 negative visits compared to seroconversion visits and additional HIV-1 RNA testing showed positive test results for only two seroconverters at the pre-seroconversion visit. Moreover, a previous sensitivity analyses of ACS data using risk behavior reported at the visit preceding the seroconversion visit did not result in a different pattern of behavioral variables associated with HIV-1 seroconversion [30]. Although we excluded visits above twelve months following the previous visit from the analyses, theoretically some seroconversions may have occurred up to eleven months prior to the first HIV-1 positive visit. However, evaluating the time interval between seroconversion visits and last seronegative visits showed that there was only one seroconverter in the ACS in which this time interval was more than nine months. These findings suggest that the results reported in the present study are expected to be robust.

A second limitation is the imputation method (last observation carried forward for missing values if the variable of the visit immediately preceding was available), being potentially inappropriate for time-sensitive risk behavior prior to seroconversion. However, complete case analyses (Additional file 1: Table S1 and S2) did not show important differences in outcomes, suggesting that the imputation approach did not introduce bias into the results.

Third, it was decided in this study to report sensitivity and specificity for every risk score at the cutoff calculated by the Youden-Index, to have a consistent approach for defining a threshold. However, the Youden-index may not be the ideal cutoff for every context. The optimal cutoff would depend on the AHI prevalence of the target population, the clinical setting and the resources available. To increase sensitivity, a lower cutoff could be used (Fig. 1a-e and Table 4). This would lower specificity, therefore a larger number of individuals would get an AHI testing advice but have a negative test result, resulting in an increase of costs.

Finally, only 112 of 175 (64%) seroconverters in the ACS reported CLAI (insertive or receptive) in the preceding six months. It is well established that CLAI is the main transmission route for acquiring HIV-1 among MSM [39] and not reporting CLAI could be due to social desirability bias, again potentially lowering the performance of the risk score. Similar rates of CLAI were described for AHI patients in a recent prospective study from the USA among MSM [40] and the data collected in both cohorts are likely consistent with clinical settings in which the risk score could be implemented as a screening tool, using clients’ self-reported behavior.

Despite these limitations, we believe that our data show that early recognition of symptoms and risk behavior in MSM could potentially enhance early diagnosis and linkage to care. Targeted screening for AHI with risk score algorithms was recently recommended to be included in clinical evaluation of high HIV-1 incidence groups [41]. Considering AHI, the Dutch HIV guidelines refer to the US guidelines [2, 42], in which it is recommended that clinicians consider a diagnosis of AHI in patients with acute retroviral syndrome and recent high risk of exposure to HIV-1. This study shows that using a risk score for AHI as a screening tool could reduce the number of individuals needing AHI testing if MSM could be targeted for AHI evaluation at the point-of-care. During a first encounter with the health care system, less than half of AHI patients were correctly diagnosed at their first visit [19, 43]. Correct and direct identification of individuals with AHI would result in the opportunity to start immediate treatment with cART. This has numerous benefits, from both a patient and a public health perspective [9, 13, 44]. Furthermore, it would offer the opportunity to provide pre-exposure prophylaxis (PrEP) to MSM in established partnerships until the index patient is virologically suppressed.

In this study, risk score **E** (including oral thrush, fever, lymphadenopathy, weight loss, gonorrhea, receptive CLAI, and more than five sexual partners) was identified as the optimal screening tool. It had good overall performance and could be incorporated in both a self-assessment (online) tool and assist clinicians with AHI screening. The online screening tool could be used by risk groups, general practitioners working with MSM, and clinicians working at STI clinics. For example, in a public health setting or STI clinic, in which STI and HIV testing are largely based on standardized protocols, the items of the risk score can be incorporated into the routine assessment of MSM. A computer-based algorithm could identify men above the threshold of 1.5 and AHI testing could be automatically offered to them. Furthermore, a self-assessment, publicly available as an online tool, could be easily completed by MSM potentially at risk for AHI or worried about acute infection; after completion of this tool they would receive a tailored advice whether or not to attend for AHI testing. Using 1.5 as a cutoff, MSM reporting oral thrush, fever, lymphadenopathy, or gonorrhea would immediately qualify for AHI screening. Summation would be required for reporting weight loss, receptive CLAI or more than five sexual partners.

## Conclusion

﻿In summary, while we initially established a risk score based on symptoms alone, we improved this algorithm by adding known predictors for HIV-1 acquisition in MSM. Screening for AHI with four symptoms (oral thrush, fever, lymphadenopathy, weight loss) and three risk factors (self-reported gonorrhea, receptive CLAI and more than five sexual partners, all in the previous six months) would lower the number of individuals requiring AHI testing and potentially enhance early diagnosis and immediate treatment.

## Additional file

Additional file 1: Tables with results of complete case analyses. (DOCX 27.2 kb)

## Abbreviations

ACS: Amsterdam Cohort Studies
AHI: Acute HIV-1 infection
aOR: Adjusted odds ratio
AUC: Area under the curve
cART: Combination antiretroviral therapy
CI: Confidence interval
CLAI: Condomless anal intercourse
GGD: Public Health Service of Amsterdam
H-TEAM: HIV Transmission Elimination AMsterdam Initiative
IQR: Interquartile range
MACS: Multicenter AIDS Cohort Study
MSM: Men who have sex with men
OR: Odd ratio
PrEP: Pre-exposure prophylaxis
ROC: Receiver operating characteristics
STI: Sexually transmitted infection
β: Beta
