# Correction to: Development and validation of a risk score to assist screening for acute HIV-1 infection among men who have sex with men

**DOI:** 10.1186/s12879-017-2805-y

**Published:** 2017-10-24

**Authors:** Maartje Dijkstra, Godelieve J. de Bree, Ineke G. Stolte, Udi Davidovich, Eduard J. Sanders, Maria Prins, Maarten F. Schim van der Loeff

**Affiliations:** 1 Academic Medical Center, Department of Infectious Diseases, University of Amsterdam, P.O. Box 227001100DE, Amsterdam, the Netherlands; 2 Public Health Service of Amsterdam, Department of Infectious Diseases, Research and Prevention, P.O. Box 22001000CE, Amsterdam, the Netherlands; 3 Amsterdam Institute for Global Health and Development, University of Amsterdam, P.O. Box 227001100DE, Amsterdam, the Netherlands; 4 Kenya Medical Research Institute, Centre for Geographic Medicine Research –Coast, P.O. Box 230, Kilifi, Kenya; 5 Nuffield Department of Clinical Medicine, University of Oxford, Oxford, OX3 7BN UK

## Correction

After publication of the original article [1] the authors noted that the following errors had occurred:

1. In the section ‘Optimising the risk score’, the word sensitivity was used instead of specificity.

This has been corrected in the sentence below:

Choosing a less stringent cutoff of 1.1 would result in sensitivity of 85.9% but lower the **specificity** to 59.3% (Fig. 1e).

2. In the ‘Discussion’ section, the incorrect value was used for weight loss. Instead of β 1.9, the correct value should be β 0.9. This figure is correct in Table 3 of the original article.

This has been corrected in the sentence below:

The optimal risk score included oral thrush (β 1.7), fever (β 1.6), lymphadenopathy (β 1.5), weight loss (β **0.9**), self-reported gonorrhea (β 1.6), receptive CLAI (β 1.1), and more than five sexual partners (β 0.9) (all in the preceding six months) (risk score E).
