# Supplementary material for: Development and validation of a risk score to assist screening for acute HIV-1 infection among men who have sex with men
Source: BMC Infect Dis. 2017 Jun 14;17:425. doi: 10.1186/s12879-017-2508-4 (PMC5471739; doi:10.1186/s12879-017-2508-4)
Supplement: Additional file 1: — Tables with results of complete case analyses. (DOCX 27.2 kb) [file 12879_2017_2508_MOESM1_ESM.docx]

**Supplementary table 1. Performance of several risk scores among participants of the Amsterdam Cohort Studies, 1984-2009, based on complete case analyses**

| *Column 1* | *2* | *3* | *4* | *5* | *6* | *7* | *8* |
| --- | --- | --- | --- | --- | --- | --- | --- |
| **Risk score** | **Cut-off^a^** | **Seroconversion visits among visits with a score of at least the cutoff^b^** | **Seroconversion visits among visits with a score below the cutoff^b^** | **Sensitivity %**  **(95% CI)** | **Specificity %**  **(95% CI)** | **Overall AUC**  **(95% CI)** | **% to be tested** |
| **Development of a risk score in Amsterdam Cohort Studies** | | | | | | | |
| **A.** As case definition^c,d^   - CLAI a condition^e^ | 2 | 80/1988 | 94/15401 | 46.0 (38.4-53.7) | 88.9 (88.4-89.4) | 0.70 (0.66-0.74) | 11.4 |
| **B.** 14 symptoms scored as beta coefficient in symptom based model^c,f^   - CLAI a condition^e^ | 0.1 | 90/2453 | 84/14936 | 51.7 (44.0-59.4) | 86.3 (85.8-86.8) | 0.70 (0.66-0.74) | 14.1 |
| **C.** 14 symptoms scored as beta coefficient in symptom based model^g,f^   - CLAI not a condition | 0.2 | 115/4399 | 59/12951 | 66.1 (58.5-73.1) | 75.1 (74.4-75.7) | 0.74 (0.70-0.78) | 25.4 |
| **D.** 14 symptoms and 3 risk factors, scored as beta coefficient in combined model^h,i^ | 1.7 | 74/3281 | 23/10835 | 76.3 (66.6-84.3) | 77.1 (76.4-77.8) | 0.83 (0.78-0.87) | 23.2 |
| **E.** 3 symptoms and 3 risk factors significant in combined model^j,k^, scored as beta coefficient | 1.5 | 68/2907 | 29/11219 | 70.1 (60.0-79.0) | 79.8 (79.1-80.4) | 0.81 (0.77-0.86) | 20.6 |

CI, confidence interval; CLAI, condomless anal intercourse. ^a^Based on Youden-index, with the exception for risk score **A** (as case definition): pre-defined cut-off was 2.  ^b^Due to missing values, the denominators of columns 3 and 4 do not add up to 17,446. Data were missing for the following variables: diarrhea, 1 missing; fatigue, 2 missings; fever, 1 missing; genital ulcers, 89 missings; nausea, 2 missings; night sweats, 1 missing; vomiting, 1 missing; weight loss, 1 missing; gonorrhea, 2546 missings; receptive condomless anal intercourse, 746 missings; >5 sexual partners, 217 missings.
^c^Due to 57 records with missing values, these risk score evaluations are based on 17,389 records.
^d^14 symptoms scored as case definition, but genital warts and oral ulcers not included due to large number of missing variables; score of 1: fatigue, headache, myalgia, nausea, night sweats, pharyngitis, or rash; score of 2: diarrhea, fever, genital ulcers, lymphadenopathy, oral thrush, oral ulcers, vomiting, or weight loss.
^e^108/175 seroconverters reported condomless anal intercourse in the preceding 6 months.
^f^In a multivariable model including 14 symptoms (table 3, column 6): diarrhea, fatigue, fever, genital ulcers, headache, lymphadenopathy, myalgia, nausea, night sweats, oral thrush, rash, sore throat, vomiting, weight loss.
^g^Due to 96 records with missing values, these risk score evaluations are based on 17,350 records.
^h^In a multivariable model including 14 symptoms and 3 risk factors (table 3, column 8): diarrhea, fatigue, fever, genital ulcers, headache, lymphadenopathy, myalgia, nausea, night sweats, oral thrush, rash, sore throat, vomiting, weight loss, gonorrhea, receptive condomless anal intercourse, >5 sexual partners.
^i^Due to 3330 records with one or more missing values, these risk score evaluations are based on 14,116 records.
^j^Fever, lymphadenopathy, oral thrush, gonorrhea, receptive condomless anal intercourse, >5 sexual partners.
^k^Due to 3320 records with one or more missing values, these risk score evaluations are based on 14,126 records.

**Supplemental table 2. Prevalence of self-reported symptoms and risk factors and their association with HIV-1 seroconversion, Amsterdam Cohort Studies, 1984-2009, based on complete case analyses**

| *Column 1* | *2* | | *3* | | *4* | *5* | *6* | *7* | *8* |
| --- | --- | --- | --- | --- | --- | --- | --- | --- | --- |
| **Symptom** | **Seroconversion visit**  **n %** | | **HIV negative visit**  **n %** | | **OR**  **(95% CI)** | **Symptom based model aOR**  **(95% CI)^a^** | **Beta coefficient^b,c^** | **Combined model aOR (95% CI)^d^** | **Beta coefficient^b,e^** |
| Total^f^ | 175 | | 17271 | |  |  |  |  |  |
| **Symptom** | | | | | | | | | |
| Diarrhea | 30 | 17.1 | 1011 | 5.9 | 3.3 (2.2-4.8) | 1.2 (0.8-2.0) | 0.2 | 1.3 (0.7-2.5) | 0.3 |
| Fatigue | 42 | 24.0 | 1866 | 10.8 | 2.6 (1.8-3.6) | 1.3 (0.8-1.9) | 0.2 | 1.6 (0.9-2.7) | 0.4 |
| Fever | 76 | 43.4 | 1623 | 9.4 | 7.2 (5.4-9.8) | 4.9 (3.5-7.0)^g^ | 1.6 | 3.6 (2.2-5.8)^g^ | 1.3 |
| Genital ulcers | 11 | 6.3 | 322 | 1.9 | 3.5 (1.9-6.5) | 2.6 (1.3-5.0)^g^ | 0.9 | 1.1 (0.3-4.0) | 0.1 |
| Headache | 18 | 10.3 | 980 | 5.7 | 1.9 (1.2-3.1) | 0.7 (0.4-1.2) | -0.4 | 0.6 (0.3-1.4) | -0.4 |
| Lymphadenopathy | 27 | 15.4 | 418 | 2.4 | 7.2 (4.7-10.9) | 3.4 (2.1-5.4)^g^ | 1.2 | 4.4 (2.4-8.1)^g^ | 1.5 |
| Myalgia | 25 | 14.3 | 869 | 5.0 | 3.1 (2.0-4.7) | 1.2 (0.7-1.9) | 0.1 | 1.4 (0.7-2.6) | 0.3 |
| Nausea | 18 | 10.3 | 526 | 3.1 | 3.6 (2.2-5.8) | 0.9 (0.4-1.8) | -0.1 | 0.5 (0.2-1.6) | -0.6 |
| Night sweats | 32 | 18.3 | 832 | 4.8 | 4.3 (3.0-6.4) | 1.2 (0.7-1.9) | 0.2 | 1.8 (1.0-3.3)^h^ | 0.6 |
| Oral thrush | 4 | 2.3 | 36 | 0.2 | 10.8 (3.8-30.7) | 5.0 (1.5-16.3)^g^ | 1.6 | 5.6 (1.1-29.6)^g^ | 1.7 |
| Rash | 11 | 6.3 | 564 | 3.3 | 2.0 (1.1-3.6) | 1.3 (0.7-2.4) | 0.2 | 0.7 (0.3-1.9) | -0.3 |
| Sore throat | 31 | 17.7 | 1316 | 7.6 | 2.6 (1.7-3.8) | 1.0 (0.7-1.6) | 0.0 | 0.8 (0.4-1.4) | -0.3 |
| Vomiting | 17 | 9.7 | 336 | 2.0 | 5.3 (3.2-8.8) | 1.9 (0.9-3.9) | 0.6 | 1.7 (0.6-4.8) | 0.5 |
| Weight loss | 11 | 6.3 | 205 | 1.2 | 5.5 (2.9-10.2) | 2.0 (1.0-4.0) | 0.7 | 2.0 (0.7-5.7) | 0.7 |
| **Risk factor** | | | | | | | | | |
| Gonorrhea^i,j^ | 12 | 6.9 | 171 | 1.0 | 7.0 (3.8-12.8) |  |  | 4.9 (2.3-10.2)^g^ | 2.0 |
| Receptive CLAI^i^ | 93 | 53.1 | 5462 | 31.6 | 2.7 (2.0-3.8) |  |  | 3.6 (2.3-5.4)^g^ | 1.3 |
| >5 sexual partners^i,k^ | 81 | 46.3 | 7599 | 44.0 | 2.3 (1.6-3.3) |  |  | 2.4 (1.5-3.6)^g^ | 0.9 |

aOR, adjusted odds ratio; CI, confidence interval; CLAI, condomless anal intercourse; OR, odds ratio ^a^14 symptoms in column 1 were included in a multivariable logistic regression model using generalized estimating equations.
^b^Natural log of the adjusted odds ratio, rounded to one decimal.
^c^Based on the adjusted odds ratios of the symptom based model (column 5).
^d^14 symptoms and 3 risk factors in column 1 were included in a multivariable logistic regression model using generalized estimating equations.
^e^Based on the adjusted odds ratios of the combined symptom and risk factor model (column 7).
^f^Due to missing values, the denominator of columns 2 and 3 are sometimes not equal to 175 respectively 17,271. Data missing for the following variables: diarrhea, 1 missing; fatigue, 2 missings; fever, 1 missing; genital ulcers, 89 missings; nausea, 2 missings; night sweats, 1 missing; vomiting, 1 missing; weight loss, 1 missing; gonorrhea, 2546 missings; receptive condomless anal intercourse, 746 missings; >5 sexual partners, 217 missings.
^g^Significantly associated with HIV-1 seroconversion.
^h^P-value = 0.06, not significantly associated with HIV-1 seroconversion.
^i^In the preceding 6 months.
^j^Self-reported.
^k^Only male sex partners, compared to ≤5 sexual partners.
